# Baseline brain imaging signs in patients with ischaemic stroke by the presence of atrial fibrillation: the ENCHANTED trial

**DOI:** 10.1007/s00415-023-11580-x

**Published:** 2023-03-20

**Authors:** Xia Wang, Shoujiang You, Zien Zhou, Candice Delcourt, Joanna Wardlaw, Grant Mair, Thompson Robinson, Xiaoying Chen, Sohei Yoshimura, Takako Torii-Yoshimura, Cheryl Carcel, Alejandra Malavera, Craig Anderson, Richard I. Lindley

**Affiliations:** 1 The George Institute for Global Health, University of New South Wales Sydney, Sydney, Australia; 2 Department of Neurology, The Second Affiliated Hospital of Soochow University, Suzhou, China; 3 Department of Radiology, Ren Ji Hospital, School of Medicine, Shanghai Jiao Tong University, Shanghai, People’s Republic of China; 4 Department of Clinical Medicine, Faculty of Medicine, Health and Human Sciences,, Macquarie University, Sydney, Australia; 5 Faculty of Medicine and Health, University of Sydney, Sydney, Australia; 6 Edinburgh Imaging, Centre for Clinical Brain Sciences, University of Edinburgh, Edinburgh, UK; 7 UK Dementia Research Institute, University of Edinburgh, Edinburgh, UK; 8 Department of Cardiovascular Sciences and NIHR Leicester Biomedical Research Centre, University of Leicester, Leicester, UK; 9 Department of Cerebrovascular Medicine, National Cerebral and Cardiovascular Center, Osaka, Japan; 10 Division of Neurology, Department of Stroke and Cerebrovascular Diseases, National Cerebral and Cardiovascular Center, Osaka, Japan; 11 Department of Neurology and Neuroscience, Nagoya City University Graduate School of Medical Science, Nagoya, Japan; 12 Department of Neurology, Royal Prince Alfred Hospital, Sydney, Australia

**Keywords:** Ischaemic stroke, Atrial fibrillation, Thrombolysis, Imaging signs

## Abstract

**Background:**

We aimed to assess the association of atrial fibrillation (AF) on outcomes in a post hoc analysis of the ENCHANTED (Enhanced Control of Hypertension and Thrombolysis Stroke Study) and how this association is modified by baseline imaging features.

**Methods:**

Inverse probability of treatment weight was used to remove baseline imbalances between those with and without AF. The primary outcome was the modified Rankin Scale (mRS) scores at 90 days. Secondary outcomes were symptomatic intracerebral haemorrhage (sICH), early neurological deterioration or death within 24 h, and death at 90 days. The logistic regression model was used to determine the associations.

**Results:**

Of the 3285 patients included in this analysis, 636 (19%) had AF at baseline. Compared with non-AF, AF was not significantly associated with an unfavourable shift of mRS (odds ratio 1.09; 95% confidence interval, 0.96–1.24), but with sICH (2.82; 1.78-4.48; IST-3 criteria), early neurological deterioration or death within 24 h (1.31; 1.01-1.70), and death (1.42; 1.12-1.79). Among patients with acute ischaemic signs (presence, extent, swelling and attenuation of acute lesions), AF was associated with the increased risk of all the poor outcomes (all *P* < 0.04 for interaction).

**Conclusions:**

We found AF increased risk of sICH, early neurological deterioration or death and death, but not unfavourable functional recovery at day 90 after thrombolysis in patients with AIS. The presence of acute ischaemic brain imaging signs at stroke presentation could be used to improve risk stratification in the presence of AF.

**Trial registration:**

The trial is registered at ClinicalTrials.gov (NCT01422616).

**Supplementary Information:**

The online version contains supplementary material available at 10.1007/s00415-023-11580-x.

## Introduction

Atrial fibrillation (AF) is the most common cause for cardioembolic stroke, which rises with age [1, 2] and is likely to be the commonest cause of ischaemic stroke in high-income countries [3]. Ischaemic stroke in the presence of AF is associated with poor functional outcomes [4–6] and a high risk of symptomatic intracerebral haemorrhage (sICH) [5, 7] after acute ischaemic stroke (AIS). Thrombolytic treatment with intravenous alteplase, when administered within 4.5 h after the onset of symptoms, is the main medical treatment for AIS [8]. Data from Virtual International Stroke Trials Archive (VISTA) of ~ 7000 patients have shown that thrombolysis derives at least equivalent treatment benefit whether in the presence or absence of AF [9]. Observational studies have produced inconsistent results: Some studies indicate that AF is associated with poorer outcomes after intravenous thrombolysis than patients without AF [10–12]. While some found no association [13], some even found an opposite association with favourable outcomes [14]. The greater risk for worse outcomes might be attributable to older age, more severe strokes at admission [13, 14], and the duration of AF [11].

Studies have also examined the link between AF and imaging factors beyond a typical vascular risk factor profile to explain the worse outcomes among patients with AF [15–19]. Patients with AF have larger brain infarctions [15], a high burden of white matter hyperintensity lesions [16, 17, 19] and silent cerebral infarctions [18]. A secondary analysis of the third International Stroke Trial (IST-3) has shown early ischaemic signs of hyperattenuated artery and pre-existing signs of atrophy and leukoaraiosis on non-contrast computed tomography (NCCT) were associated with reduced independence and increased risk of sICH after thrombolysis [20]. However, there is scant literature regarding how those imaging signs modified the association between AF and clinical outcomes among AIS after thrombolytic treatment. We aimed to explore those interactions using data from the dose arm of the Enhanced Control of Hypertension and Thrombolysis Stroke Study (ENCHANTED). The hypothesis was the association between AF and clinical outcomes was modified by those early ischaemic or pre-existing imaging signs.

## Methods

### Participants

The ENCHANTED trial was an international, multi-centre, prospective, randomised, open-label, blinded endpoint trial, the details of which are outlined elsewhere [21–23]. In brief, 3310 patients with a clinical diagnosis of AIS confirmed on brain image and fulfilling local criteria for thrombolysis treatment, including symptom onset within 4.5 h, and were randomised in the dose arm of the ENCHANTED trial to receive low- (0.6 mg/kg; 15% as bolus, 85% as infusion over 1 h) or standard-dose (0.9 mg/kg; 10% as bolus, 90% as infusion over 1 h) alteplase. The study protocol was approved by the appropriate ethics committee at each participating centre, and written informed consent was obtained from each patient or an appropriate surrogate. The study is registered with Clinicaltrials.gov (number NCT01422616).

### Procedures

Key demographic and clinical characteristics were recorded at the time of enrolment. Stroke severity was measured using the NIHSS at baseline, 24 h, and at day 3 (or earlier on discharge from the hospital). Uncompressed digital images of all baseline and follow-up digital CT, MRI and angiogram images were collected in DICOM format on a CD-ROM identified only with the patient’s unique study number and uploaded by a special purpose-built web-based system for central analysis at The George Institute for Global Health. All brain scans with an intracranial haemorrhage were reviewed by at least two independent assessors, blind to clinical data, treatment, and date and sequence of the scan, using MIStar version 3.2 (Apollo Medical Imaging Technology, Melbourne, Victoria, Australia). Assessors graded any haemorrhage as intracerebral, subarachnoid, intraventricular, subdural or other; sICH being graded across all standard definitions [21–23]. Our imaging assessment protocol was derived from the IST3 methodology [20], details including the definition of the imaging variables have been described elsewhere (Supplementary file 1) [20, 24].

The primary outcome in these analyses was the ordinal analysis of the modified Rankin Scale (mRS) at 90 days. Secondary efficacy outcomes included early neurologic deterioration (≥ 4 points decline in NIHSS scores) or death within 24 h, mRS scores of 2–6 on the mRS, mRS scores of 3–6 on the mRS, and death at day 90 alone. The safety outcome was sICH defined according to several criteria used in other studies.

### Statistical analysis

We used the dose arm of the ENCHANTED trial as an observational cohort study. The characteristics of patients with AF were expected to substantially differ from those of patients without AF. To generate a comparable data set, we calculated a propensity score to estimate the individual probability of a patient having AF. The following variables (significant variables of < 0.05 from univariate analysis) were used to calculate propensity scores (age, sex, ethnic groups [Asian vs non-Asian], premorbid mRS [0 vs 1], a history of the acute coronary syndrome, or other heart diseases, smoking, medication history of antihypertensives, or antiplatelet, or anticoagulant, or lipid-lowering drugs, baseline NIHSS, systolic blood pressure, heart rate, time from onset to randomisation, randomised treatment (low-dose vs standard-dose alteplase), leukoaraiosis, Visible infarct, hypoattenuation, swelling, hyperattenuated artery, atrophy, and leukoaraiosis. The inverse probability of treatment weighting (IPTW), instead of propensity score matching to maximise sample size, was used as the primary strategy to adjust for baseline imbalances [25]. As the recommended method of propensity score matching is to match either 1 or 2 untreated subjects to each treated subject [26]. Consequently, we would lose a significant proportion of patients. Data balancing was examined using an absolute standardised difference in covariate means [27]. The distributions of baseline covariates were fairly well balanced by applying propensity scores; the absolute standardised differences after IPTW were within an acceptable margin of 0.1 (Supplementary Fig. 1). Stabilized weights [28] were used to reduce the variance of the estimated effect of the presence of AF and were incorporated into logistic regression models to determine associations of the presence of AF and outcomes.

The association of the presence of AF on outcomes was determined using logistic regression models, and the heterogeneity of the association across subgroups by imaging factors was estimated by adding an interaction term to the statistical models. Data were reported as odds ratios (OR) and 95% confidence intervals (CI). Two-sided *P* values are reported, with *P* < 0.05 considered statistically significant. SAS version 9.3 (SAS Institute, Cary, NC) was used for analyses [28].

## Results

3285 patients (1243 [37.8%] female) of mean age 66.6 (SD 12.8) from the dose arm of the ENCHANTED trial were included in the analysis. Table 1 shows that patients with AF [636(19%)] were significantly more likely to be older, female, of non-Asian ethnicity, disabled before the stroke, and with a history of co-morbidities (coronary artery disease, other cardiac diseases, and hypercholesterolaemia), and concomitant therapies (antihypertensives, anticoagulation, antiplatelet, and statin). As expected, patients with AF were more likely to have a cardioembolic stroke, and thus strokes of greater severity. We observed patients with AF were more likely to have hyperattenuated artery, atrophy, and leukoaraiosis, whereas less likely to have visible infarct, hypoattenuation and swelling. The baseline characteristics are well balanced between patients with AF and without AF after propensity score (Figure SI).

**Table 1:** Baseline Characteristics by a history of atrial fibrillation

	No AF	AF	*P* value
Time from stroke onset to randomisation (hrs),	2.7 (2.1–3.5)	2.5 (1.8–3.3)	< 0.0001
Age (years)	65.0 (12.7)	72.8 (11.4)	< 0.0001
≥ 80	307/2649 (11.6)	164/ 636 (25.8)	< 0.0001
Female	946/2649 (35.7)	297/ 636 (46.7)	< 0.0001
Asian	1701/2649 (64.2)	374/ 636 (58.8)	0.011
Clinical features			
Systolic BP (mmHg)	149.9 (19.5)	146.5 (20.6)	0.000
Diastolic BP (mmHg)	84.6 (12.5)	85.0 (14.4)	0.704
Heart rate (beats per minute)	77.2 (13.6)	86.8 (19.3)	< 0.0001
GCS score	15.0 (14.0–15.0)	14.0 (12.0–15.0)	< 0.0001
NIHSS score	8.0 (5.0–12.0)	13.0 (8.0–18.0)	< 0.0001
Medical History			
Hypertension	1650/2649 (62.3)	413/ 636 (64.9)	0.215
Previous stroke	461/2649 (17.4)	127/ 636 (20.0)	0.130
Coronary artery disease	343/2649 (12.9)	136/ 636 (21.4)	< 0.0001
Other heart disease (valvular or other)	159/2649 (6.0)	76/ 636 (11.9)	< 0.0001
Diabetes mellitus	529/2649 (20.0)	115/ 636 (18.1)	0.282
Hypercholesterolaemia	426/2649 (16.1)	129/ 636 (20.3)	0.011
Current smoker	686/2646 (25.9)	84/ 635 (13.2)	< 0.0001
Pre-stroke function (mRS)			
No symptoms	2207/2648 (83.3)	465/ 635 (73.2)	< 0.0001
No significant disability	441/2648 (16.7)	170/ 635 (26.8)	
Medication at time of admission			
Antihypertensive agents	1149/2649 (43.4)	347/ 636 (54.6)	< 0.0001
Warfarin anticoagulation	35/2646 (1.3)	47/ 636 (7.4)	< 0.0001
Aspirin or other antiplatelet agents	532/2646 (20.1)	220/ 636 (34.6)	< 0.0001
Glucose-lowering agents	332/2646 (12.5)	83/ 636 (13.1)	0.732
Statin or other lipid-lowering agent	454/2646 (17.2)	161/ 635 (25.4)	< 0.0001
Final diagnosis at time of hospital separation			
Large artery occlusion due to significant atheroma	1136/2649 (42.9)	133/ 636 (20.9)	< 0.0001
Small vessel or perforating vessel lacunar disease	638/2649 (24.1)	35/ 636 (5.5)	
Cardioembolism	227/2649 (8.6)	413/ 636 (64.9)	
Others	25/2649 (0.9)		
ASPECTS			0.002
0–4	145/2317 (6.3)	18/ 591 (3.0)	
5–7	237/2317 (10.2)	42/ 591 (7.1)	
8–10	1935/2317 (83.5)	531/ 591 (89.8)	
Early ischaemic signs			
Visible infarct^a^	821/2313 (35.5)	165/ 590 (28.0)	0.0006
Hypoattenuation	707/2313 (30.6)	139/ 590 (23.6)	0.0008
Large lesion	90/2304 (3.9)	26/ 581 (4.5)	0.533
Swelling	817/2317 (35.3)	164/ 591 (27.7)	0.0006
Hyperattenuated artery	344/2317 (14.9)	146/ 591 (24.7)	< 0.0001
Pre-existing signs			
Atrophy	1396/2317 (60.3)	447/ 591 (75.6)	< 0.0001
Leukoaraiosis	725/2317 (31.3)	225/ 591 (38.1)	0.0017
Old infarct	821/2317 (35.4)	199/ 591 (33.7)	0.423
Randomised low-dose treatment	1315/2649 (49.6)	330/ 636 (51.9)	0.309

Data are *n* (%), mean (SD), or median (IQR), The *P* values are based on Chi-square, *T* test, or Wilcoxon signed-rank test

*NIHSS* The National Institutes of Health Stroke Scale, *BP* blood pressure, *GCS* Glasgow Coma Scale, *mRS* modified Rankin scale, *CT* computed tomography, *MRI* magnetic resonance imaging

^a^Represents any of hypoattenuation, early ischaemic lesion size, swelling, and hyperattenuated artery

**Fig. 1:**
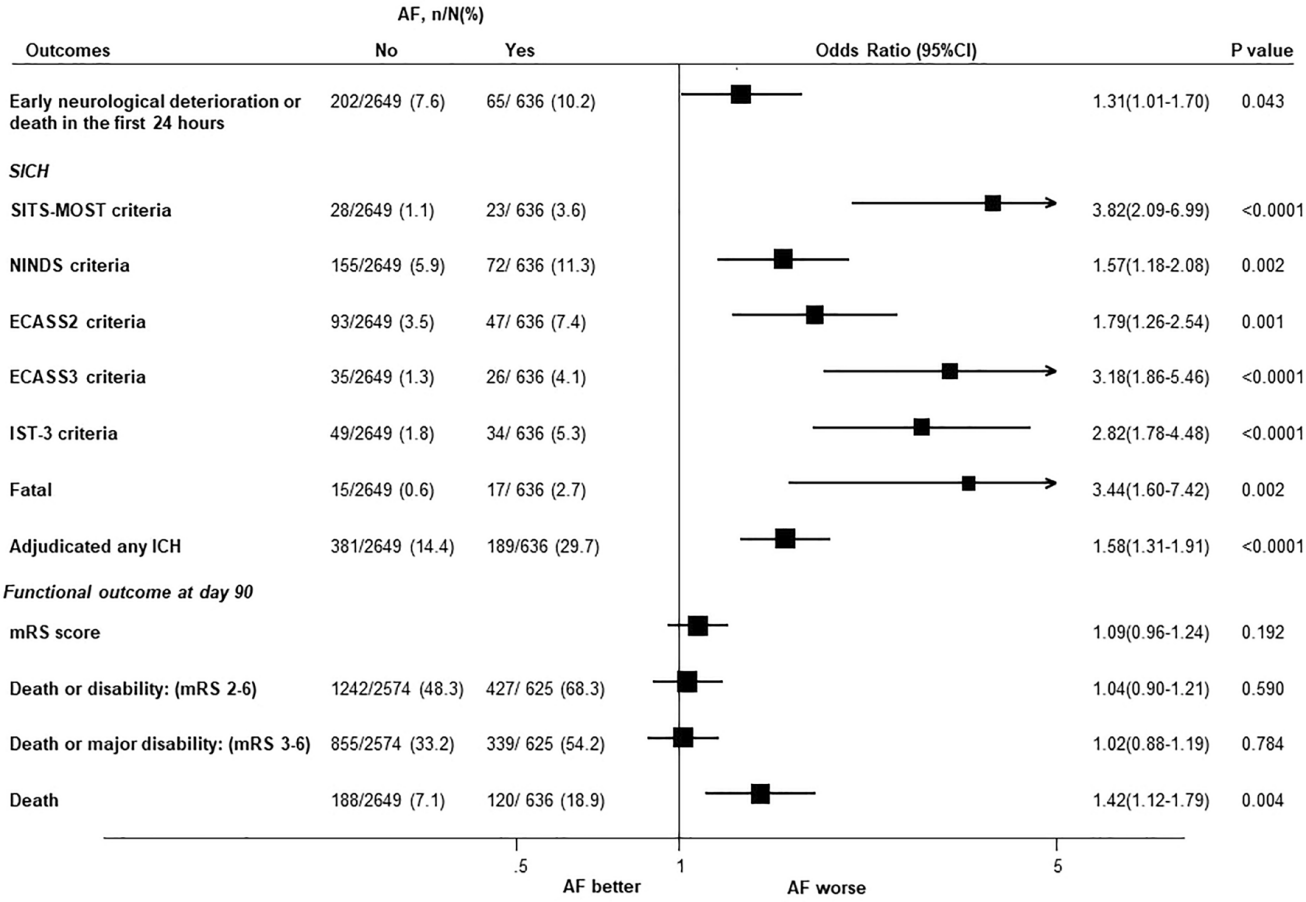
The association between a history of atrial fibrillation and key clinical outcomes. *AF* atrial fibrillation, *CI* confidence interval, *ECASS* The European Cooperative Acute Stroke Study, *mRS* modified Rankin Scale, *NINDS* The National Institute of Neurological Disorders and Stroke, *IST-3* The third International Stroke Trial; SICH symptomatic intracerebral haemorrhage, *SITS-MOST* the Safe Implementation of Thrombolysis in Stroke-Monitoring Study. The propensity score was generated from a model with sex, ethnic groups (Asian vs non-Asian), premorbid mRS (0 vs 1), medication history of antihypertensives, a history of acute coronary syndrome, or other heart diseases, smoking, antiplatelet, anticoagulant, lipid-lowering drugs, age, baseline NIHSS, SBP, HR, time from onset to randomisation, randomised treatment (low-dose vs standard-dose alteplase), hypoattenuation, swelling, hyperattenuated artery, atrophy, early ischaemic lesion size, and leukoaraiosis

Compared with non-AF, patients with AF were not at increased risk of an unfavourable shift of mRS at day 90 (OR: 95% CI 1.09: 0.96–1.24) (*P* = 0.192 for proportional odds assumption test) (Fig. 1). However, AF was associated with ~ 1.5-fold increased risk of death (1.42: 1.12–1.79), and ~ fourfold of sICH (3.82: 2.09–6.99) defined by the Safe Implementation of Thrombolysis in Stroke-Monitoring Study (SITS-MOST) criteria and ~ threefold (2.82: 1.78–4.48) defined by IST-3 criteria. The consistent significant association with sICH was seen across a broad range of criteria (all *P* < 0.002) (Fig. 1). AF was also associated with a ~ 1.5-fold increased risk of early neurological deterioration or death in the first 24 h (1.31: 1.01–1.70).

We found that acute ischaemic signs (presence, extent, swelling and attenuation of acute lesions) significantly modified the association between AF and ordinal mRS at day 90 (all *P* ≤ 0.005 for interaction), death (all *P* ≤ 0.002 for interaction), and sICH (any adjudicated sICH, all p ≤ 0.004 to 0.037 for interaction) (Table 2). Among patients presenting with any of those acute ischaemic signs, AF was associated with increased risk of poor outcomes for an unfavourable shift of mRS, any adjudicated sICH, and death, respectively).

**Table 2:** The interaction of imaging factors and the association between a history of AF and clinical outcomes after propensity score

	MRS at 90 days		Any adjudicated sICH		death	
	OR (95% CI)	*P* interaction	OR (95% CI)	*P* interaction	OR (95%CI)	*P* interaction
Prior anticoagulant		0.999		0.489		0.274
Prior antiplatelet		0.839		0.055		**0.002**
No					1.80 (1.36–2.39)	
Yes					0.78 (0.50–1.21)	
Acute ischaemic signs						
Early ischaemic lesion size		**0.005**		**0.002**		**0.002**
None visible	0.95 (0.81–1.11)		1.28 (1.00–1.64)		0.83 (0.60–1.15)	
Small	3.29 (1.93–5.59)		1.85 (0.85–4.03)		8.15 (3.44–19.30)	
Medium	1.24 (0.93–1.66)		2.32 (1.61–3.35)		3.32 (1.43–3.76)	
Large	2.59 (1.22–5.47)		2.97 (1.24–7.14)		2.32 (0.93–5.81)	
Hypoattenuation		**0.0003**		**0.006**		** < 0.0001**
No	0.96 (0.82–1.12)		1.31 (1.03–1.66)		0.81 (0.59–1.12)	
Yes	1.62 (1.26–2.07)		2.29 (1.67–3.16)		3.07 (2.09–4.50)	
Swelling		**0.0003**		**0.037**		** < 0.0001**
No	0.95 (0.81–1.11)		1.36 (1.06–1.75)		0.86 (0.62–1.19)	
Yes	1.58 (1.25–1.99)		2.06 (1.53–2.76)		2.61 (1.82–3.73)	
Hyperattenuated artery		0.498		0.780		0.162
Visible infarct^a^		**0.0002**		**0.027**		** < 0.0001**
No	0.95 (0.80–1.10)		1.34 (1.04–1.73)		0.86 (0.62–1.20)	
Yes	1.59 (1.27–2.01)		2.08 (1.55–2.80)		2.57 (1.80–3.67)	
The number of acute ischaemic signs^b^		**0.0002**		**0.016**		** < 0.0001**
0	0.95 (0.80–1.12)		1.20 (0.91–1.58)		0.93 (0.64–1.34)	
1	0.86 (0.54–1.37)		2.27 (1.23–4.18)		0.61 (0.30–1.26)	
2	2.16 (1.07–4.37)		1.70 (0.75–3.87)		0.59 (0.16–2.24)	
3	1.55 (1.16–2.08)		2.86 (1.90–4.30)		4.13 (2.48–6.88)	
4	1.67 (1.05–2.68)		1.54 (0.89–2.67)		2.06 (1.11–3.83)	
Pre-existing signs						
Atrophy		0.065		0.192		0.221
Leukoaraiosis		0.418		**0.008**		** < 0.0001**
No			1.90 (1.51–2.39)		2.22 (1.64–3.01)	
Yes			1.09 (0.77–1.53)		0.64 (0.43–0.95)	
Old infarct		**0.022**		0.626		0.877
No	0.99 (0.84–1.16)					
Yes	1.39 (1.11–1.74)					

*CI* confidence interval, *mRS* modified Rankin Scale, *OR* odds ratio, *sICH* symptomatic intracerebral haemorrhage

^a^Represents any of hypoattenuation, early ischaemic lesion size, swelling, and hyperattenuated artery

^b^Acute ischaemic signs include hypoattenuation, early ischaemic lesion size, swelling, and hyperattenuated artery

There are means the significance of P for interaction between AF and acute ischaemic signis on clinical outcomes

## Discussion

This post hoc analysis of the ENCHANTED trial indicated that the presence of AF was associated with increased risk of sICH, mortality, and early neurological deterioration in thrombolysed patients with AIS, considering the differences explained by traditional vascular risk factors and imaging features. However, no significant differences for functional recovery at day 90 were observed between patients with and without AF. Moreover, we found that acute ischaemic brain imaging signs significant modified the association between AF and poor outcomes. Compared with non-AF, AF increases the risk of all poor outcomes, including poor functional recovery, sICH, and death in the subgroup of patients with acute ischaemic signs.

Our analyses have produced a robust association derived from a large population recruited from a wide range of health care settings, using a broad range of different classifications of ICH, rigorous outcome assessment. We have adjusted for both traditional vascular risk factors and imaging features, including early ischaemic and pre-existing signs. Our results are consistent with most previous observations studies showing a high odds of sICH and mortality in patients with AF after thrombolysis [10–12]. Patients with AF were also more likely to develop early neurological deterioration or death within 24 h [10]. Inconsistent results produced by some small studies [13, 14] could be explained by limited sample size, different classifications of sICH and incomplete confounder adjustment.

A secondary analysis of the third International Stroke Trial (IST-3) has shown the predictive significance of early ischaemic and pre-existing signs for sICH and functional outcomes after thrombolysis [20]. Given the different AIS mechanisms for patients with and without AF, we hypothesised that the direction/magnitude of those associations was different between patients with and without AF. This analysis found a higher risk of poor outcomes with AF than non-AF among patients with all acute ischaemic signs (presence, extent, swelling and attenuation of acute lesions). The acute ischaemic signs of an early ischaemic lesion, hypoattenuation and swelling reflect the middle cerebral artery’s occlusion with insufficient cerebral collateral circulation supply and are associated with larger infarct volume [29, 30]. Previous studies have shown that patients with AF were more likely to have greater volumes of baseline hypoattenuation, greater infarct growth, greater final infarct volume for sudden main trunk occlusion, and less developed cerebral collateral circulation [31, 32]. Those mechanisms support our results in explaining the more frequent haemorrhagic transformation and poor outcomes among patients with AF after AIS [32, 33]. Our finding in line with recently thrombectomy registry study indicated that AF patients exposed to intravenous thrombolysis before mechanical thrombectomy had increased haemorrhagic complications without improved functional outcomes, in contrast with non-AF patients [34].

Our findings may have relevant implications for clinical practice. First, our data help to explain and understanding the higher risk of haemorrhagic transformation and poor outcomes after thrombolysis in AIS patients with AF by acute ischaemic signs on NCCT. Second, our results suggest direct thrombectomy may suitable treatment strategy for AF patients with acute ischaemic signs on NCCT, which usually reflect large vessel occlusion.

This study’s strength includes the large sample size and recruitment from different healthcare settings, a prospective design with high rates of follow-up, and detailed systematically measured baseline NCCT signs on clinical outcomes in patients with and without AF. Weaknesses include the important point that we have used the ENCHANTED trial as a cohort study, and therefore, despite our efforts to determine the independent significance of associations, we cannot presume causality in such observational analysis, and such multiple post hoc testing raises the potential for chance associations. First, most patients had a mild-to-moderate neurological deficit. Therefore, the present findings may not be generalisable to patients with more severe stroke. Second, magnetic resonance imaging is more sensitive and clearer than NCCT in detecting the early ischaemic and pre-existing signs, while only a few patients had a baseline MRI scan. Third, we lack data on cerebral microbleeds, which is the important marker of cerebral small vessel disease. Fourth, the proportion of patients who received mechanical thrombectomy was fairly low in the ENCHANTED study. Therefore, the present findings may not be generalisable to patients with large vessel occlusion and who receive mechanical thrombectomy treatment.

## Conclusion

In summary, we confirm that AF was significantly associated with an increased risk of sICH, early neurological deterioration and mortality but not functional recovery at 90 days in thrombolysed patients with AIS. The presence of acute ischaemic signs on NCCT is a significant effect modifier for the association between AF and poor outcomes and could be used to improve risk stratification when the presence of AF.

## Supplementary Information

Below is the link to the electronic supplementary material.

Supplementary file1 (PDF 73 KB)

## Data Availability

Individual deidentifed participant data used in these analyses will be shared by request from any qualified investigator following approval of a protocol and signed data access agreement via the Research Offce of The George Institute for Global Health, Australia.
