# Supplementary material for: Baseline brain imaging signs in patients with ischaemic stroke by the presence of atrial fibrillation: the ENCHANTED trial
Source: J Neurol. 2023 Mar 20;270(5):2567–75. doi: 10.1007/s00415-023-11580-x (PMC10130001; doi:10.1007/s00415-023-11580-x)
Supplement: Supplementary file 1 — Supplementary file1 (PDF 73 KB) [file 415_2023_11580_MOESM1_ESM.pdf]

Baseline brain imaging signs in ischaemic patients by the presence of atrial fibrillation: the ENCHANTED trial

Wang X et al

Supplemental File

The definition of imaging variables
SFigure I Standardised mean differences after propensity score

The defnitions of imaging variables

The presence and degree of *hypoattenuated tissue* was defined as either mild (grey matter attenuation equal to normal white matter) or severe (grey and white matter attenuation less than normal white matter) [1].

We graded ischaemic *lesion swelling* on a widely validated 7-point ordinal scale [2] but due to small numbers in categories 4 to 6, these were grouped into a single category of 'severe effacement'.

The extent of acute ischaemic lesions was classified in two ways: with the IST3 method, which includes the whole brain where the score for infarct extent includes all arterial territories, [2] and with the Alberta Stroke Program Early CT Score (ASPECTS) [3] which focuses only on the middle cerebral artery (MCA) territory, although the version of ASPECTS used in IST3 and for our study allowed additional scoring of abnormal anterior cerebral artery (ACA) and posterior cerebral artery (PCA) territories. Thus, we used the IST3 score as the primary measure of ischaemic lesion size in analyses, condensing the full IST3 lesion extent score into four groups: (1) small lesions, as lacunar, small cortical, small cerebellar, less than half of brainstem or less than half of the ACA or PCA territory; (2) medium lesions, classified as striatocapsular, the anterior or posterior half of the peripheral MCA territory, or more than half the ACA or PCA territory; (3) *large lesions*, defined as the whole of the peripheral MCA territory or all the MCA territory and (4) very large lesions, which comprised the whole MCA and PCA territory, all the MCA and ACA territory or all three territories. For ischaemia within the ACA circulation, the corresponding scores on ASPECTS were 8-10, 5-7 and 0–4 for small, medium and large or very large lesions on the IST3 score [4].

The presence or absence, and location of any *hyperattenuated artery* was acordingg to Kharitonova et al study [5].

We classified *cerebral atrophy* as none, moderate or severe, when compared against standard examples as used previously [6].

The presence and severity of *leucoaraiosis* on CT [7] or MRI [8]were recorded using validated scales.

The location of *old infarcts*, including cortical, lacunar, border zone and brainstem or cerebellar were according to Wardlaw et al study [2].

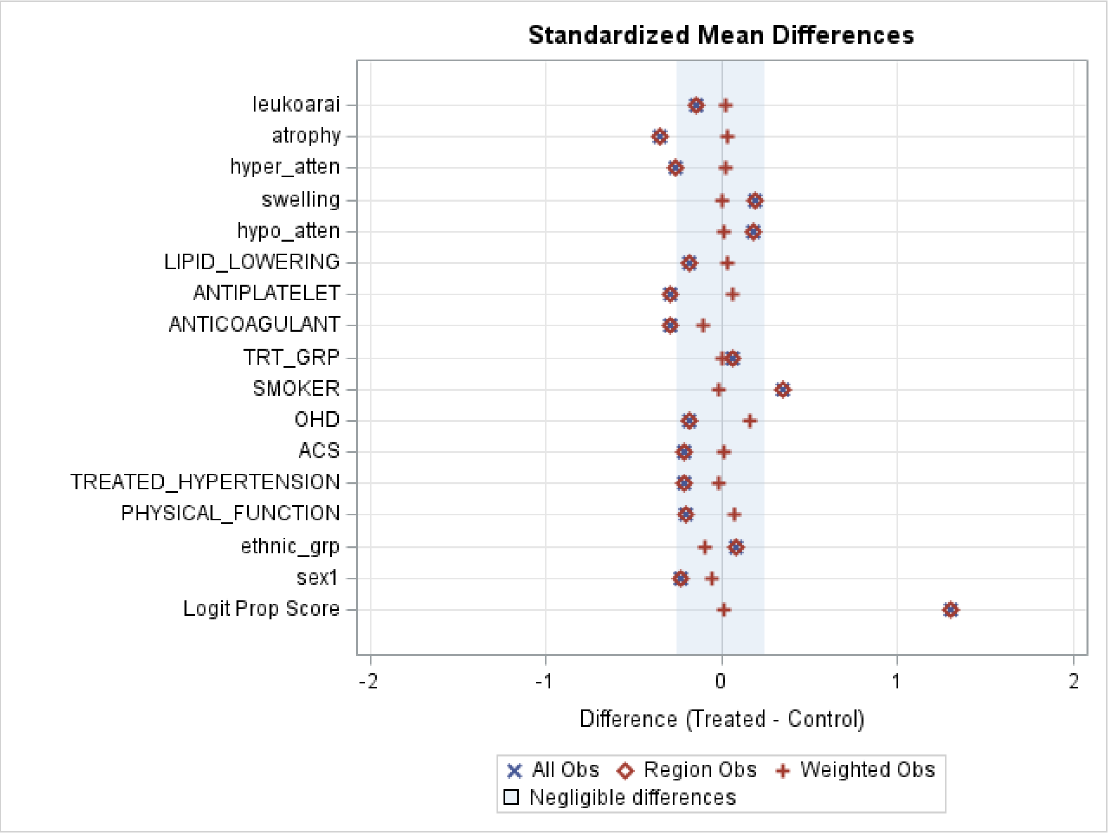

SFigure I Standardised mean differences after propensity score

1. Wardlaw JM, Mielke O. Early signs of brain infarction at CT: observer reliability and outcome after thrombolytic treatment--systematic review. Radiology 2005;235:444-453.
2. Wardlaw JM, Sellar R. A simple practical classifcation of cerebral infarcts on CT and its interobserver reliability. AJNR Am J Neuroradiol 1994;15:1933-1939.
3. Barber PA, Demchuk AM, Zhang J, et al. Validity and reliability of a quantitative computed tomography score in predicting outcome of hyperacute stroke before thrombolytic therapy. The Lancet 2000;355:1670-1674.
4. Wardlaw JM, West TM, Sandercock PAG, et al. Visible infarction on computed tomography is an independent predictor of poor functional outcome after stroke, and not of haemorrhagic transformation. J Neurol Neurosurg Psychiatry 2003;74:452-458.
5. Kharitonova T, Ahmed N, Thorén M, et al. Hyperdense middle cerebral artery sign on admission CT scan--prognostic signifcance for ischaemic stroke patients treated with intravenous thrombolysis in the safe implementation of thrombolysis in Stroke International Stroke Thrombolysis Register. Cerebrovasc Dis 2009;27:51-59.
6. Farrell C, Chappell F, Armitage PA, et al. Development and initial testing of normal reference Mr images for the brain at ages 65-70 and 75-80 years. Eur Radiol 2009;19:177-183.
7. van Swieten JC, Hijdra A, Koudstaal PJ, et al. Grading white matter lesions on CT and MRI: a simple scale. J Neurol Neurosurg Psychiatry 1990;53:1080-1083.
8. Fazekas F, Chawluk JB, Alavi A, et al. Mr signal abnormalities at 1.5 T in Alzheimer’s dementia and normal aging. AJR Am J Roentgenol 1987;149:351-356.
